# Effect of Electronic Cigarette Vapour Exposure on Ca^2+^- and cAMP-Dependent Ion Transport in Human Airway Epithelial Cells

**DOI:** 10.1007/s00408-025-00805-7

**Published:** 2025-03-18

**Authors:** Ya Niu, Chung-Yin Yip, Ke-wu Pan, Judith Choi-Wo Mak, Wing-Hung Ko

**Affiliations:** 1 School of Biomedical Sciences, The Chinese University of Hong Kong, Hong Kong, China; 2 Department of Pharmacology & Pharmacy, The University of Hong Kong, Hong Kong, China; 3 The Chinese University of Hong Kong, Rm. 607A, Lo Kwee-Seong Integrated Biomedical Sciences Building, Shatin, N.T., Hong Kong, China

**Keywords:** E-cigarette, Airway epithelia, Ion transport, CFTR, Ca^2+^, cAMP

## Abstract

**Purpose:**

The popularity of electronic cigarettes (e-cigarettes) has grown exponentially over the past few years, and teenagers now prefer them to tobacco cigarettes. We determined whether exposure to e-cigarette vapour (e-vapour) adversely affects ion transport using human airway epithelial cell lines 16HBE14o- and Calu-3 and well-differentiated primary human bronchial epithelial cells (HBEs).

**Methods:**

We concurrently measured fluorescent signals and short-circuit current (*I*_*SC*_), an indicator of electrogenic ion transport, in polarised epithelia. The P2Y receptor-mediated signalling pathway was used to induce an increase in intracellular calcium concentration ([Ca^2+^]_i_) and *I*_*SC*_. We used a single-polypeptide fluorescence resonance energy transfer reporter based on exchange proteins directly activated by cAMP (Epac) to measure forskolin-induced changes in cAMP and *I*_*SC*_.

**Results:**

We compared the effects of e-vapour to those of traditional cigarette smoke (CS) on the human airway cell models. In all three cell types, e-vapour, similar to CS, significantly reduced agonist-induced increases in Ca^2+^ or cAMP signalling and *I*_*SC*_. However, reductions in the epithelial electrolyte transport activities did not correlate with any changes in the protein levels of various ion channels and transporters.

**Conclusion:**

Our data suggest that e-vapour is not harmless and causes ion transport dysfunction similar to CS, thereby predisposing e-cigarette users to vaping-induced lung injury.

## Introduction

Cigarette smoke (CS) is a well-characterised major risk factor for lung diseases. Normal airway surface hydration requires active ion transport by the airway epithelium, which is adversely affected by CS. Cyclic AMP (cAMP) and Ca^2+^ are important signalling molecules that regulate transepithelial Cl^−^ transport. CS impairs both cAMP- and Ca^2+^-dependent Cl^−^ secretion across the epithelia, resulting in dehydration of the airway surface and impairment of mucociliary clearance [1, 2]. Moreover, CS hinders the expression of the cystic fibrosis transmembrane conductance regulator (CFTR) gene, protein, and function in vitro, and cigarette smokers acquire CFTR deficiency in their nasal respiratory epithelium. Therefore, cigarette-induced diseases, such as chronic bronchitis, may be associated with acquired CFTR deficiency [1]. An electrophysiological study showed that 30-min exposure to CS increased the permeability of rabbit tracheal epithelium mounted in an Ussing chamber to ions [3]. As a result, smoking exposes the body to potentially harmful chemicals, which may lead to irreversible and harmful effects and initiate pro-carcinogenic processes.

Throughout the last decade, the popularity of e-cigarettes, often seen as an alternative to smoking, has exploded, and teenagers now prefer them to tobacco cigarettes. However, the effects of e-cigarettes on respiratory health have not been thoroughly evaluated. E-cigarettes comprise propylene glycol, glycerol, flavour compounds, and nicotine (Nic)-containing liquids that are inhaled via the respiratory tract. In human airway epithelial cells, e-cigarette exposure may result in ion channel dysfunction, particularly for ion transport that depends on Ca^2+^ or the P2Y receptor. Furthermore, e-cigarette exposure disrupts the barrier function of the respiratory epithelium [4, 5], exacerbates respiratory syncytial virus infection [5], induces airway inflammation and mucus hypersecretion [6], and impairs mucociliary clearance [7]. The inhalation of Nic in an aerosol form hinders CFTR-mediated anion transport, reduces periciliary liquid depth, and impairs mucociliary movement in Ferret’s airways, thereby affecting the mucociliary clearance defence against inhaled pathogens and particulates [7].

The air–liquid interface (ALI) culture technique provides a mucociliary model for studying lung biology that recapitulates normal airway epithelium biology in vitro. Previously, we found that human bronchial epithelium cultured at an ALI contained different cell types, including basal, ciliated, and secretory cells, providing a model that simulates the biological properties of normal bronchial epithelium in vitro [8] and can be used to characterise several properties, such as active ion transport and transepithelial electrical resistance. In this study, we compared the effects of e-cigarette vapour (e-vapour) and CS on human airway epithelial function, specifically ion transport activities, in two human airway epithelial cell lines, 16HBE14o- and Calu-3, and well-differentiated primary human bronchial epithelial cells (HBEs).

## Materials and Methods

### Cell Culture

16HBE14o- cells and Calu-3 cells (ATCC, VA, USA) were maintained in a minimal essential medium as described previously [9]. For simultaneous measurement of [Ca^2+^]_i_, or cAMP and short-circuit current (*I*_*SC*_), cells were seeded onto Transwell-COL membranes (0.4-µm pore size; Costar, Cambridge, MA, USA) with a culture area of 0.1 cm^2^ or 0.2 cm^2^. As previously described, we cultured primary HBEs (ScienCell Research Laboratories, CA, USA; Cat #3210, Lot #6457) from a single healthy donor at the ALI to produce pseudostratified mucociliary epithelia [8]. In brief, HBEs were grown in PneumaCult-Ex Plus Expansion Medium (#05040, STEMCELL, WA, USA). Subcultured HBEs were seeded on poly-L-lysine–coated Transwell permeable inserts. The cells were expanded before confluence using an expansion medium. Cells were air-exposed by removing the apical medium, and the basolateral medium was replaced with PneumaCult-ALI Maintenance Medium (#05001, STEMCELL, WA, USA) on day 0 (D0). A pseudostratified layer was obtained on ALI-D28. Transepithelial electrical resistance measurement was performed as described previously [8]. Similarly, both 16HBE14o- and Calu-3 cells were cultured under ALI conditions for 14 days post-confluence.

### Exposing HBEs, 16HBE14o-, or Calu-3 Epithelia to e-Vapour or CS

ALI-cultured primary HBEs, 16HBE14o-, or Calu-3 epithelia were exposed to (i) e-vapour (30 or 70 watts [W]), (ii) CS (Camel cigarettes with filter), or (iii) air (control) using a Buxco Smoke Generation and Delivery System (Data Sciences International, MN, USA) similar to that described by Manevski et al. [10]. In the system, an electrically powered e-cigarette device (SMOK^®^ X-Priv, Shenzhen IVPS Technology, SZ, PRC) generates e-vapour at various wattages. The system was also connected to an aerosol concentration measurement instrument (MicroDust Pro, Casella, Bedford, UK). Real-time monitoring of the total particulate matter (TPM) concentration generated by the vaping protocol of the smoking machine was performed before the e-vapour was delivered to the cultured cells inside the chamber. The concentration of the TPM generated at 30 and 70 W was relatively constant at 268.7 ± 49.9 and 833.3 ± 65.3 mg/m^3^ (*n* = 6), respectively. Three brands of e-liquid used were Reds Apple (Red; CA, USA), Saucy (SAUCY; CA, USA), and Ice (ICE; NRT, Japan). The e-liquids contained ~ 65% propylene glycol (PG), ~ 35% vegetable glycerine (VG), and food flavours with or without 20 mg/mL Nic. The SAUCY e-liquid had a Kiwi fruit, apple, and menthol flavour. Reds Apple was flavoured with berries, and Ice was flavoured with litchi. We used a high-puff volume and frequency protocol [11] comprising a 55-ml puff drawn over 3 s at 30-s intervals. Cells were exposed to e-vapour for 12 cycles prior to fluorescence and electrophysiological measurements. The puffing profile for CS exposure was the same as that used for e-vapour generation, ensuring that cells were exposed to the same amount of Nic. The total Nic content of two cigarettes consumed every 12 cycles was 1.6 mg. In the same 12 cycles, 0.08 mL of an e-liquid containing 20 mg/mL Nic was consumed, which is equivalent to 1.6 mg of Nic. The Nic content of e-cigarettes typically ranges from 3 to 36 mg/mL. The e-cigarettes of recent generations contain much more Nic (up to 60 mg/mL), usually in a salt form, so that Nic is delivered to the brain at a rate comparable to that of cigarette smoking [12]. Consequently, our e-liquid had a Nic content of 20 mg/mL. Based on the 65/35 ratio of PG to VG in e-liquids, we prepared a vehicle control of 65% PG and 35% VG (with/without 20 mg/mL Nic). Liquid Nic (≥ 99% (GC)) was added to the e-liquid to achieve the desired concentration. Following exposure, the epithelia were immediately transferred to a miniature Ussing chamber to measure intracellular calcium or cAMP levels and *I*_*SC*_.

### Simultaneous Measurements of [Ca^2+^]_i_ or cAMP and *I*_*SC*_

ATP- or UTP-induced Ca^2+^ signalling and anion secretion were measured simultaneously in polarised epithelia as described previously [9]. For *I*_*SC*_ measurement, in brief, the monolayers were mounted in a miniature Ussing chamber and bathed in normal bicarbonate-buffered Krebs–Henseleit (KH) solution of the following composition (mM): NaCl, 117; NaHCO_3_, 25; KCl, 4.7; MgCl_2_, 1.2; KH_2_PO_4_, 1.2; CaCl_2_, 2.5; D-glucose, 11. The pH of the solution was 7.4 when the solution was bubbled with 5% CO_2_/95% O_2_. A basolateral-to-apical Cl^−^ gradient favourable for apical Cl^−^ exit was established across the monolayers by changing the apical KH solution to a low Cl^−^ solution. In the low (10 mM) Cl^−^ solution, NaCl, KCl, CaCl_2_, and MgCl_2_ were replaced isosmotically with Na-gluconate, K-gluconate, Ca-gluconate, and MgSO_4_, respectively. The potential difference was clamped to 0 mV, and *I*_*SC*_ was simultaneously measured using a voltage clamp amplifier (VCC MC6; Physiologic Instruments, San Diego, CA, USA). A voltage pulse of 1 mV was applied periodically, and the resultant change in current was used in the calculation of the transepithelial resistance by Ohm’s law.

Imaging experiments used to measure intracellular cAMP and [Ca^2+^]_i_ were conducted as described previously [13]. Real-time changes in the cAMP levels in living cells were monitored using Cyan Fluorescent Protein (CFP)-Epac-Yellow Fluorescent Protein (YFP), an Epac-based polypeptide fluorescence resonance energy transfer (FRET) reporter [14]. The monolayers were transfected with the Epac-based cAMP sensor for 2 days and then used for imaging experiments. FRET imaging experiments were performed with the constant perfusion of the KH solution at the apical and basolateral sides of the epithelia at 37 °C using an inverted microscope (Olympus IX70, Center Valley, PA, USA) with a 20 × /0.6 NA water immersion objective. The MetaFluor Imaging System with a FRET module (Molecular Devices, LLC, Sunnyvale, CA, USA) was used to control image acquisition. The cells were sequentially excited at 436 nm. The emission light was split by the Photometrics DV2 two-channel simultaneous imaging system (Photometrics, Tucson, AZ, USA), and emissions from CFP (470/30 nm filter) and YFP (FRET; 535/30 nm filter) were captured using a scientific CMOS camera (pco.edge 5.5; PCO AG, Kelheim, Germany). The acquired fluorescence images were background-corrected, and real-time changes in cAMP levels were represented by normalised CFP/FRET emission ratios. An increase in cAMP levels corresponded to an increase in the FRET ratio.

For [Ca^2+^]_i_ measurement, the cells were loaded with 3 μM Fura-2-AM and excited at 340 and 380 nm; and Fura-2 emission (> 510 nm) was recorded, and the changes in [Ca^2+^]_i_ were monitored by Fura-2 340/380 ratiometric imaging. A rise in [Ca^2+^]_i_ correspondsed to an increase in the Fura-2 ratio. All signals were digitised, and data analysis was performed using the MetaFluor Imaging Software (v.7.5 with FRET module).

### Western Blotting

16HBE14o-, Calu-3, and primary HBE monolayers were exposed to e-vapour for three consecutive days as described above and samples were collected 8 h after the third exposure to e-vapour for western blot analysis, which was performed as described previously [15]. For immunoblotting, the filter screen and filter paper were soaked in pre-cooled transfer buffer in advance. The gel and membrane (0.45 μm, PVDF) were cut according to the size of the target protein, soaked in methanol for 30 s for activation, and then soaked in pre-cooled transfer buffer. The transfer splint was installed according to the sandwich structure (filter screen–filter paper–gel–membrane–filter paper–filter screen) and correctly placed in the transfer tank according to the current direction. The transfer was conducted in an ice–water bath at a constant current of 300 mA for 2–4 h. After the transfer was complete, the PVDF membrane was taken out immediately, washed in a TBST washing solution for 1 min, and then blocked in a blocking solution at room temperature for 1 h. The primary antibodies were TMEM16A (#BP1-60076, 1:2000, NOVUS), NKCC1 (#8351, 1:2000, CST), KCNQ1 (#ab84819, 1:1000, Abcam), CFTR (#sc-376683, 1:1000, Santa Cruz, TX, USA), KCNN4 (#23271-1-AP, 1:2000, Proteintech, IL, USA), and β-ACTIN (#sc-8432, 1:3000, Santa Cruz). The secondary antibodies were goat anti-mouse (#31430, 1:10,000, Invitrogen) and goat anti-rabbit (#31460, 1:10,000, Invitrogen, MA, USA) HRP-conjugated secondary antibodies. Protein bands were visualised using the chemiluminescence substrate ECL (#1705061, Bio-Rad, CA, USA) and captured using a Bio-Rad ChemDoc Imaging System. Protein band intensities were quantified using the ImageJ programme and normalised to β-ACTIN.

### Chemicals

The membrane-permeant acetoxymethylester (AM) form of Fura-2 was obtained from Molecular Probes (Eugene, OR, USA). ATP, UTP, Nic, and forskolin were obtained from Sigma-Aldrich (St. Louis, MO, USA). The laboratory reagents for general use were obtained from Sigma-Aldrich (St. Louis, MO, USA). All tissue culture reagents were obtained from Invitrogen.

### Statistical Analysis

All data are expressed as means ± S.E.M., and values of *n* refer to the number of experiments in each group. Experimentally induced changes (∆) in Fura-2 ratios, FRET ratios, and *I*_*SC*_ were measured at the peak of a response with subtraction of the values measured immediately prior to stimulation. Comparison of the means was performed using Student’s t-tests or one-way ANOVA (followed by post hoc tests) as appropriate using Graph Pad Prism 8 software (Prism, San Diego, CA, USA). A *p*-value of ≤ 0.05 was considered significant.

## Results

### Effect of e-Vapour on Ca^2+^- and cAMP-Dependent *I*_*SC*_ in 16HBE14o- Cells

**Fig. 1:**
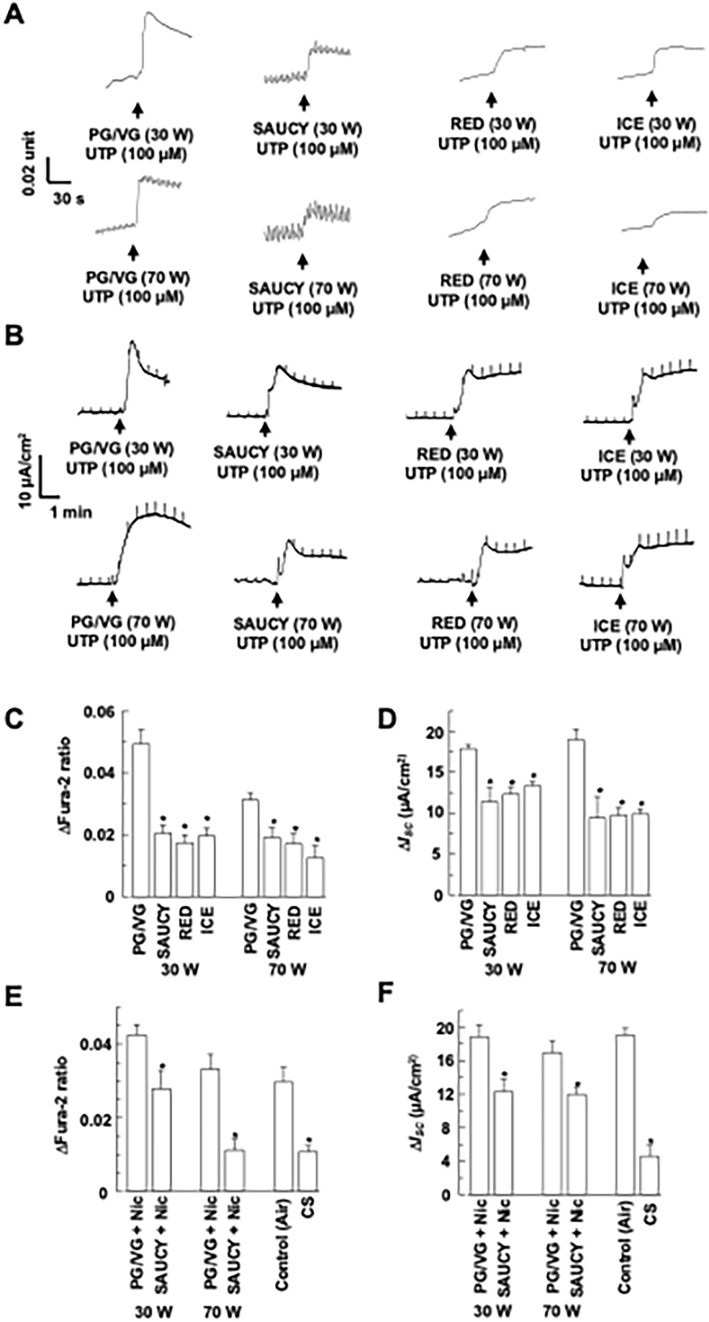
Effect of e-vapour, e-vapour with nicotine (Nic), and cigarette smoke (CS) on UTP-induced changes in [Ca.^2+^]_i_ and *I*_*SC*_ of 16HBE14o- epithelia. (**a**, **b**) Epithelia were treated with SAUCY, RED, or ICE e-vapours at 30 or 70 W and then stimulated with apical UTP (100 μM) to induce changes in Fura-2 ratios (**a**) and *I*_*SC*_ (**b**), which were monitored simultaneously. Transient pulses shown in (**b**) are the current responses to intermittent voltage pulses at 1 mV. Summarised data are shown in (**c**) and (**d**). (**e, f**) Epithelia were treated with SAUCY + Nic at 30 or 70 W or CS and then stimulated with apical UTP (100 μM) to induce changes in Fura-2 ratios (**e**) and *I*_*SC*_ (**f**), which were monitored simultaneously. Data are presented as means ± S.E.M. for 4–10 individual epithelia. *p < 0.05 compared with the control (PG/VG or air)

To determine the effect of e-vapour and CS on Ca^2+^-dependent Cl^−^ secretion in 16HBE14o- epithelia, we measured UTP-induced changes in [Ca^2+^]_i_ and *I*_*SC*_ simultaneously after exposure of cells to e-vapour. UTP increases [Ca^2+^]_i_ via activation of the P2Y_2_ or P2Y_4_ purinergic receptors, followed by an increase in *I*_*SC*_ due to Cl^−^ secretion in 16HBE14o- epithelia [9]. Preliminary experiments demonstrated that amiloride (100 μM), a sodium channel blocker that inhibits epithelial sodium channel activity, did not inhibit agonist-induced *I*_*SC*_ increases in 16HBE14o- and Calu-3 epithelia; therefore, amiloride was not included in the experiments described below. Epithelia were exposed to 12 puffs of SAUCY, RED, or ICE e-vapour using a smoking machine. Figure 1a, b show representative tracings for each experiment. UTP (100 μM) increased Fura-2 ratios (Fig. 1a) and *I*_*SC*_ (Fig. 1b) in control epithelia (PG/VG). Exposure to three different e-vapours (SAUCY, RED, and ICE) consistently caused a significant reduction in the UTP-induced increase of [Ca^2+^]_i_ and *I*_*SC*_. Figure 1c, d summarise the effects of e-vapour generated at 30 W or 70 W on changes in Fura-2 ratios and *I*_*SC*_ induced by UTP. The UTP-induced [Ca^2+^]_i_ and *I*_*SC*_ changes were inhibited and there were no differences among the three brands of e-liquids (Figs. 1c, d; *n* = 4–10). In the next series of experiments, exposure of 16HBE14o- epithelia to SAUCY + Nic at 30 W or 70 W also inhibited UTP-induced [Ca^2+^]_i_ and *I*_*SC*_ responses vs. the control (PG/VG) containing the same amount of Nic (Fig. 1e, f; *n* = 4–10). Similarly, CS from one cigarette inhibited the UTP-induced [Ca^2+^]_i_ and *I*_*SC*_ responses (Fig. 1e, f).

**Fig. 2:**
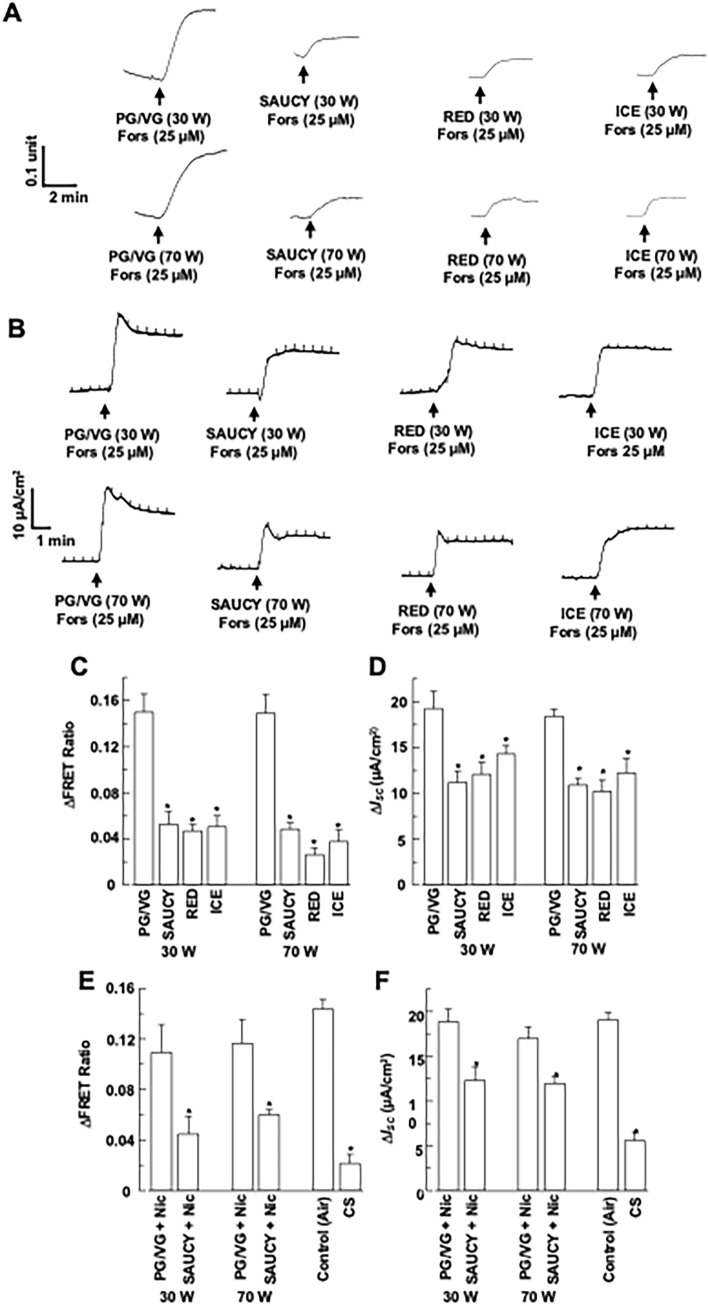
Effect of e-vapour, e-vapour with nicotine (Nic), and cigarette smoke (CS) on forskolin-induced changes in cAMP levels and *I*_*SC*_ of 16HBE14o- epithelia. (**a, b**) Epithelia were treated with SAUCY, RED, or ICE e-vapours at 30 or 70 W and then stimulated with apical forskolin (25 μM) to induce changes in FRET ratios (**a**) and *I*_*SC*_ (**b**), which were monitored simultaneously. Transient pulses shown in (**b**) are the current responses to intermittent voltage pulses at 1 mV. Summarised data are shown in (**c**) and (**d**). (**e, f**) Epithelia were treated with SAUCY + Nic at 30 or 70 W or CS and then stimulated with apical forskolin (25 μM) to induce changes in FRET ratios (**e**) and *I*_*SC*_ (**f**), which were monitored simultaneously. Data are presented as means ± S.E.M. for 4–10 individual epithelia. *p < 0.05 compared with the control (PG/VG or air)

We next repeated the treatments of 16HBE14o- epithelia stimulated with forskolin, an adenylate cyclase activator that elevates intracellular cAMP levels and Cl^−^ secretion [16]. E-vapour generated from the three e-liquid brands significantly inhibited the forskolin-mediated increase in FRET ratios (Fig. 2a) and *I*_*SC*_ (Fig. 2b) vs. the control (PG/VG). Figure 2c, d summarise the data and show that the three brands of e-liquid have no differing inhibitory effects (*n* = 4–10). In addition, the inhibition of forskolin-induced increases in FRET ratios or *I*_*SC*_ were similar in 16HBE14o- epithelia exposed to SAUCY + Nic at 30 W and 70 W and to CS (Fig. 2e, f; *n* = 4–10).

### Effect of e-Vapour on Ca^2+^- and cAMP-Dependent *I*_*SC*_ in Calu-3 Cells

**Fig. 3:**
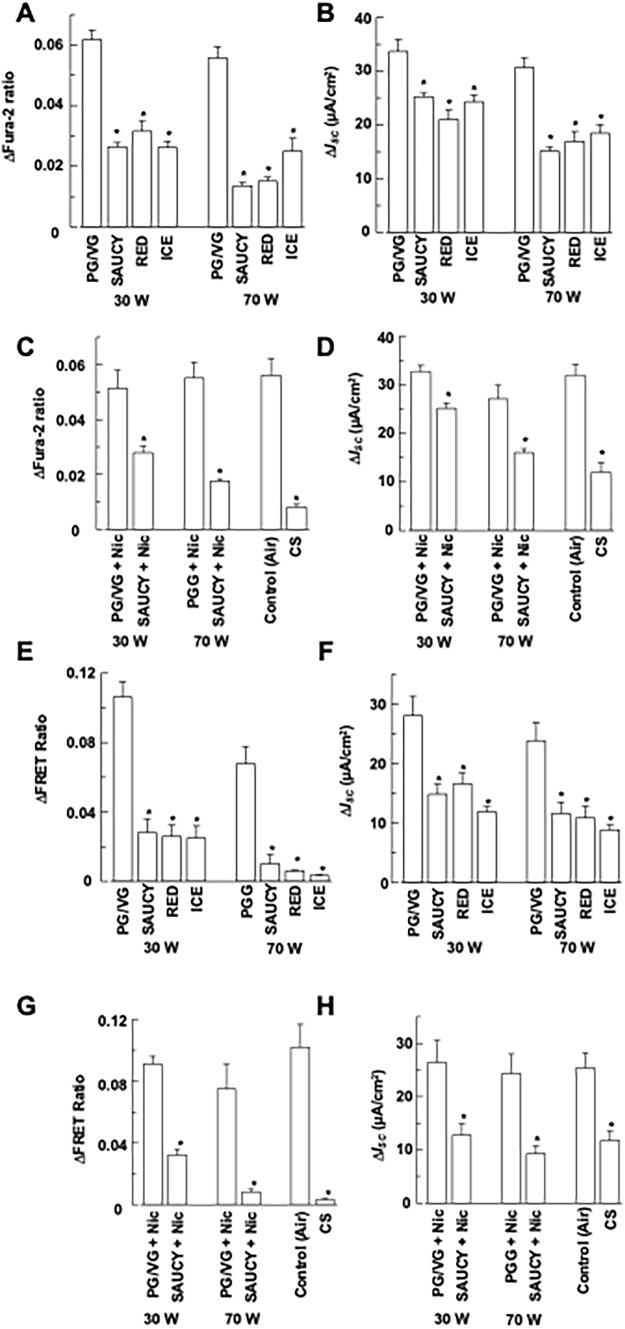
Effect of e-vapour, e-vapour with nicotine (Nic), and cigarette smoke (CS) on UTP-induced changes in [Ca.^2+^]_i_ and *I*_*SC*_ or forskolin- induced changes in cAMP levels and *I*_*SC*_ of Calu-3 epithelia. (**a, b**) Epithelia were treated with SAUCY, RED, or ICE e-vapours at 30 or 70 W and then stimulated with apical ATP (100 μM) to induce changes in Fura-2 ratios (**a**) and *I*_*SC*_ (**b**), which were monitored simultaneously. (**c, d**) Epithelia were treated with SAUCY, RED, or ICE e-vapours at 30 or 70 W and then stimulated with apical forskolin (25 μM) to induce changes in FRET ratios (**c**) and *I*_*SC*_ (**d**), which were monitored simultaneously. (**e, f**) Epithelia were treated with SAUCY + Nic at 30 or 70 W or CS and then stimulated with apical ATP (100 μM) to induce changes in Fura-2 ratios (**e**) and *I*_*SC*_ (**f**), which were monitored simultaneously. (**g, h**) Epithelia were treated with SAUCY + Nic at 30 or 70 W or CS and then stimulated with apical forskolin (25 μM) to induce changes in FRET ratios (**g**) and *I*_*SC*_ (**h**), which were monitored simultaneously Data are presented as means ± S.E.M. for 4–10 individual epithelia. *p < 0.05 compared with the control (PG/VG or air)

Similar experiments were performed in Calu-3 epithelia as conducted in 16HBE14o- epithelia. Since P2Y_2_ and P2Y_4_ receptors are not expressed in Calu-3 cells [17], ATP (100 μM) was used to stimulate an increase in [Ca^2+^]_i_ in Calu-3 cells, presumably via activation of P2Y_1_ receptors [18]. The treatment of Calu-3 epithelia with e-vapour generated from three different e-liquids (SAUCY, RED, and ICE) at 30 or 70 W inhibited ATP-induced [Ca^2+^]_i_ (Fig. 3a) and *I*_*SC*_ (Fig. 3b) responses (*n* = 4–10) when compared to the control (PG/VG). Exposing the Calu-3 epithelia to e-vapour, SAUCY + Nic, or CS (Fig. 3c, d; *n* = 4–10) caused a reduction in ATP-induced [Ca^2+^]_i_ and *I*_*SC*_ responses.

Next, Calu-3 epithelia were treated with forskolin (25 μM) to study the effects of e-vapour exposure on cAMP-dependent Cl^−^ secretion. Exposure of Calu-3 epithelia to e-vapour generated at 30 W or 70 W using the smoking machine inhibited the forskolin-mediated increase in cAMP levels (Fig. 3e) and *I*_*SC*_ (Fig. 3f) responses (*n* = 4–10). Similar inhibitory effects on forskolin-induced increases in cAMP levels (Fig. 3g) or *I*_*SC*_ (Fig. 3h) responses were observed when Calu-3 epithelia were exposed to SAUCY + Nic or CS (*n* = 4–10).

### Effect of e-Vapour on Ca^2+^- and cAMP-Dependent *I*_*SC*_ in Primary HBEs

**Fig. 4:**
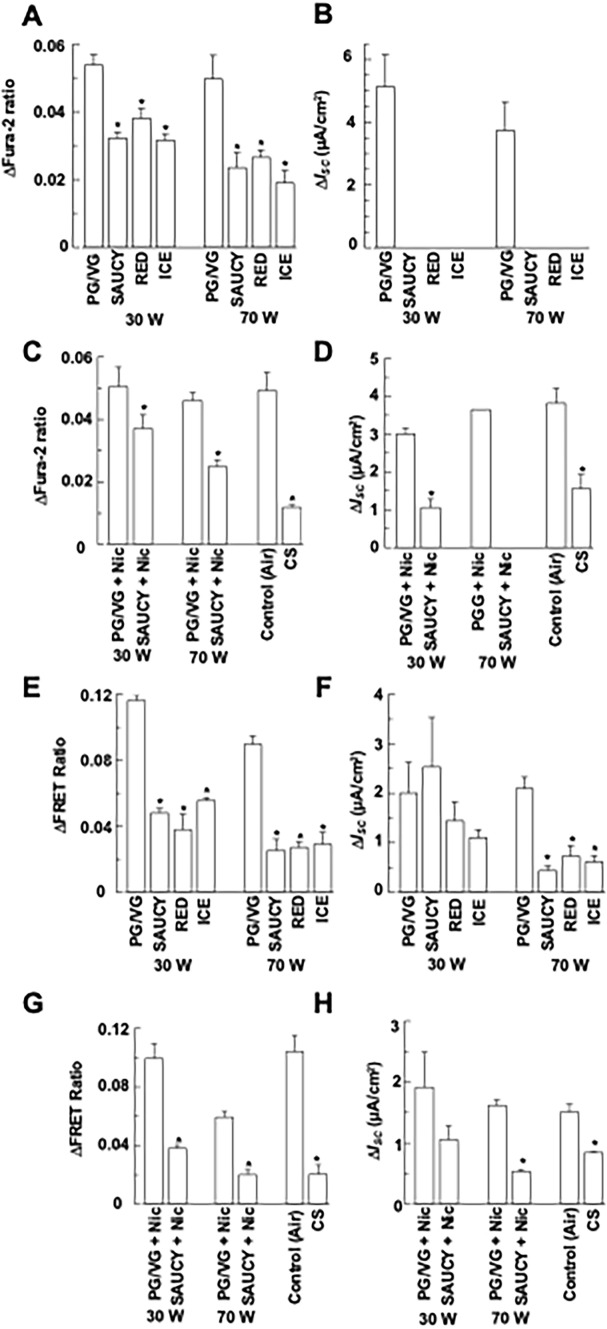
Effect of e-vapour, e-vapour with nicotine (Nic), and cigarette smoke (CS) on UTP-induced changes in [Ca.^2+^]_i_ and *I*_*SC*_ or forskolin- induced changes in cAMP levels and *I*_*SC*_ of primary HBEs. (**a, b**) Epithelia were treated with SAUCY, RED, or ICE e-vapours at 30 or 70 W and then stimulated with apical UTP (100 μM) to induce changes in Fura-2 ratios (**a**) and *I*_*SC*_ (**b**), which were monitored simultaneously. (**c, d**) Epithelia were treated with SAUCY, RED, or ICE e-vapours at 30 or 70 W and then stimulated with apical forskolin (25 μM) to induce changes in FRET ratios (**c**) and *I*_*SC*_ (**d**), which were monitored simultaneously. (**e, f**) Epithelia were treated with SAUCY + Nic at 30 or 70 W or CS and then stimulated with apical UTP (100 μM) to induce changes in Fura-2 ratios (**e**) and *I*_*SC*_ (**f**), which were monitored simultaneously. (**g, h**) Epithelia were treated with SAUCY + Nic at 30 or 70 W or CS and then stimulated with apical forskolin (25 μM) to induce changes in FRET ratios (**g**) and *I*_*SC*_ (**h**), which were monitored simultaneously. Data are presented as means ± S.E.M. for 4–10 individual epithelia. *p < 0.05 compared with the control (PG/VG or air)

E-vapour exposure at 30 W and 70 W significantly reduced UTP-induced [Ca^2+^]_i_ responses as measured by Fura-2 ratios in primary HBEs (in the presence of 100 μM amiloride in the apical perfusion solution) (Fig. 4a; *n* = 4–10). The UTP-stimulated increase in *I*_*SC*_ in primary HBEs was very sensitive to the different e-vapours, and no change in *I*_*SC*_ was detected (i.e. zero UTP-induced increase in *I*_*SC*_) (Fig. 4b; *n* = 4–10). Exposing primary HBEs to SAUCY + Nic or CS reduced the UTP-induced increase in Fura-2 ratios (Fig. 4c) and *I*_*SC*_ (Fig. 4d) responses (*n* = 4–10). Again, no increase in UTP-induced *I*_*SC*_ was observed when the epithelia were treated with SAUCY + Nic at 70 W.

To measure the effect of e-vapour and CS on cAMP-dependent *I*_*SC*_, we performed similar experiments, but the primary HBEs were stimulated with forskolin. Exposing primary HBEs to e-vapour attenuated the forskolin-induced increase in FRET ratios at both 30 W and 70 W (Fig. 4g; *n* = 4–10). Exposing primary HBE to e-vapour at 70 W, but not 30 W, decreased the forskolin-induced *I*_*SC*_ (Fig. 4h; *n* = 4–10). SAUCY + Nic or CS also reduced the forskolin-stimulated increase in FRET ratios (Fig. 4g) and *I*_*SC*_ (Fig. 4h) in primary HBEs (*n* = 4–10).

### Effect of e-Vapour and CS on Protein Expression Patterns of Various Ion Channels and NKCC1 in Primary HBEs

**Fig. 5:**
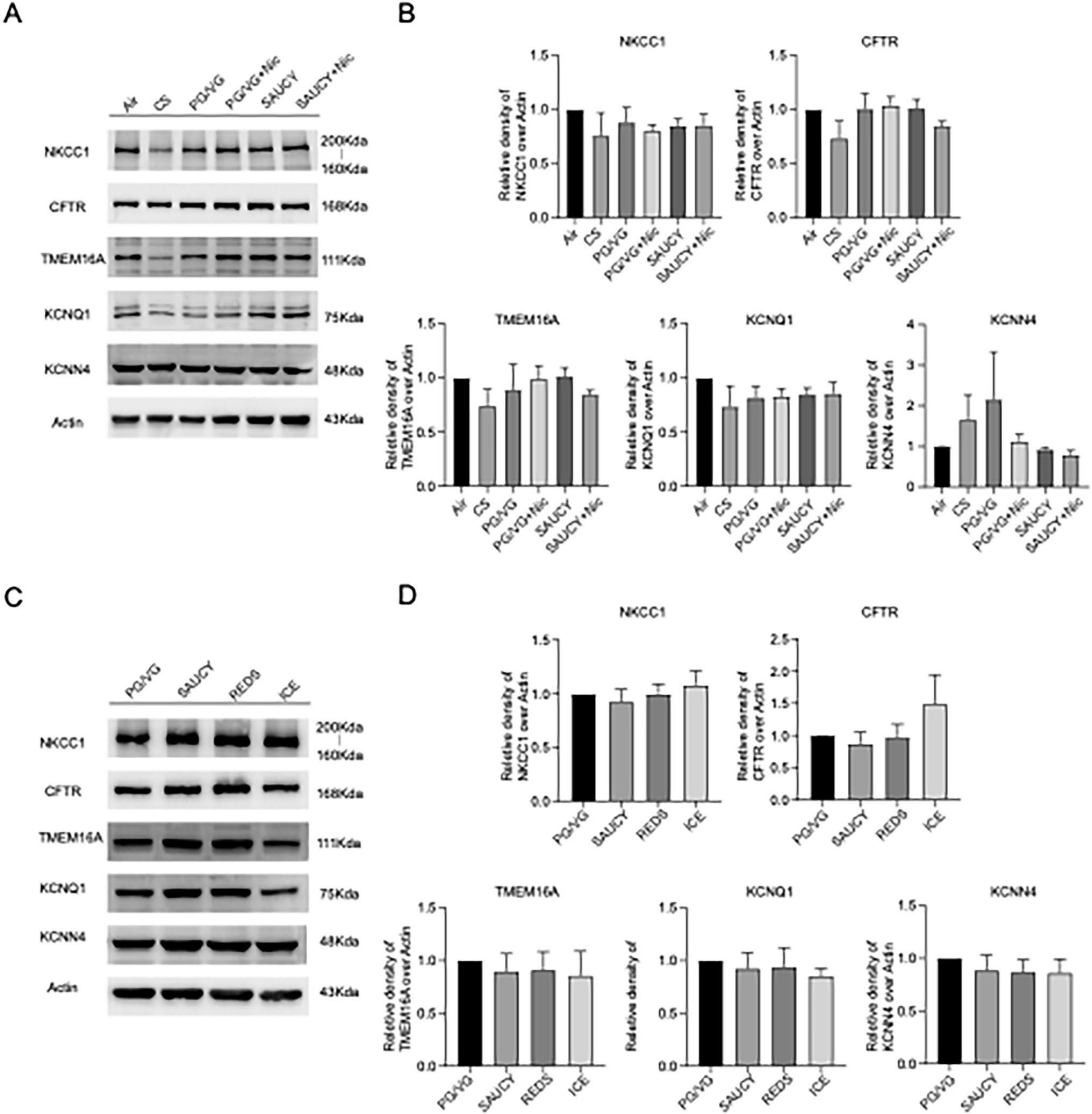
Effect of air, cigarette smoke (CS), PG/VG, e-vapour (SAUCY, REDS, ICE), and SAUCY with or without nicotine (Nic) on protein levels of Mucin 5AC, NKCC1, and various ion channels in primary HBEs. Representative western blot images (**a**, **c**) showing the effect of air, CS, PG/VG, different e-vapours (with or without Nic) on protein levels of Mucin 5AC, NKCC1, and various epithelial ion channels (CFTR, TMEM16A, KCNQ1, and KCNN4) in primary HBEs. (**b**, **d**) Summary showing the density of each band relative to the control after normalisation to β-actin (actin). Data are presented as means ± S.E.M. for 3–4 separate epithelia. ANOVA (with post hoc test) was used to determine significant differences between the two groups, but no significant differences were found

We measured the protein expression levels of ion channels or transporters involved in Ca^2+^- and cAMP-dependent Cl^−^ secretion by western blotting after various treatments of primary HBEs (*n* = 3–4). Representative western blots of CFTR (cystic fibrosis transmembrane conductance regulator), TMEM16A (transmembrane protein 16A), KCNQ1 (cAMP-dependent K^+^ channel), KCNN4 (Ca^2+^-activated K^+^ channel), and NKCC1 (Na^+^/K^+^/2Cl^−^ cotransporter) isolated from primary HBEs under various treatment conditions are shown (Fig. 5a, c). The relative changes in expression for each protein after cell treatment with normalisation to β-ACTIN did not support our hypothesis that the reduction in *I*_*SC*_ was due to a change in the expression of proteins related to transepithelial ion transport (Fig. 5b, d). Thus, the data suggest that altered expression of ion channels or transporter proteins does not explain the inhibitory effect of e-cigarettes on *I*_*SC*_.

## Discussion

We found that e-cigarettes strongly affected ion transport function, one of the most important functions of human airway epithelia in three ALI-cultured airway epithelial cells, 16HBE14o-, Calu-3, and primary HBEs. The 16HBE14o- cells derived from the human airway epithelium [19] retain the differentiated epithelial morphology and functions of airway surface epithelial cells, providing a promising in vitro cell model for the study of airway epithelial ion transport [20–22], barrier function [23, 24], inflammation [25, 26], cell biology [27, 28], and signalling pathways [29, 30]. The human airway serous glandular epithelial cell line Calu-3, an in vitro model of the airway epithelium, is used in CS and e-vapour research [31] because its physiology is similar to cells in vivo [32, 33]. Compared to submerged cultures, the in vitro ALI culture technique better replicates the normal airway epithelium for studying lung biology [34]. Submerged cultures are completely covered by e-liquid diluted in culture medium, which is non-physiological because the airway lining fluid is normally exposed to air on the apical surface. We showed previously that ALI cultures of primary HBEs fully differentiated into ciliated cells, goblet cells, basal cells, and club cells [8]. Using ALI cultures exposed to e-vapour using a smoking machine, we determined the effects on ion transport functions for three airway epithelial models in an improved physiologically relevant in vitro model. Using a technically demanding approach that measures two parameters (Ca^2+^ or cAMP and *I*_*SC*_) simultaneously in polarised epithelia allowed us to better correlate the inhibitory effects of e-vapour on Ca^2+^- and cAMP-dependent ion transport activities. E-vapour exposure in three human airway epithelial models resulted in a similar reduction in Ca^2+^ and cAMP signals, followed by a decrease in transepithelial anion secretion. These results suggest that e-vapour affects airway epithelial ion transport and could result in respiratory problems related to ion transport dysfunction.

Hydration of the normal airway surface depends on active ion transport processes in the highly water-permeable airway epithelia [35]. Ion transport-dependent hydration helps to maintain the thickness and composition of the airway surface liquid, which in turn affects airway mucus clearance [36], a vital innate defence mechanism of the lung against disease. Abnormal airway surface liquid volume, salt content, or mucus clearance compromises airway immunity and predisposes the airway to respiratory diseases and lung infections [37]. By inducing ion transport dysfunction, e-cigarettes compromise normal airway epithelial function, which can result in severe lung disease and infection.

A recent study using ALI-cultured Calu-3 cells suggests that the damaging effects of e-vapour, including decreased transepithelial resistance and increased release of lactate dehydrogenase, depend on the flavour [31]. However, we found that e-vapour inhibition of Ca^2+^- and cAMP-dependent ion transport was similar for all three brands of e-liquids and was independent of Nic. We found similar inhibitory effects on ion transport in epithelia exposed to CS, confirming the detrimental effect of traditional cigarettes on epithelial ion transport. Thus, e-cigarettes and traditional cigarettes cause similar reductions in human airway epithelial ion transport. Our data indicate that e-cigarettes are harmful, and the damaging effects of aerosols on ion transport are comparable to traditional cigarettes.

Increased cAMP activates Cl^−^ secretion via luminal CFTR Cl^−^ channels [38]. In addition, cAMP also increases basolateral K^+^ conductance via K_v_LQT_1_-type K^+^ channels (e.g. KCNQ1) that hyperpolarise the membrane and increase the driving force for apical Cl^−^ exit [39]. Ca^2+^-activated Cl^−^ channels (CaCCs; TMEM16A) are also involved in Cl^−^ secretion [40]. Increases in [Ca^2+^]_i_ concentrations lead to the opening of CaCCs and basolateral SK4-type K^+^ channels (e.g. KCNN4) [39] that also drive the exit of Cl^−^ through apical Cl^−^ channels (i.e. CFTR and CaCCs). Therefore, Ca^2+^ and cAMP are the two major signal transduction molecules that regulate airway ion transport. Here, we show that e-cigarettes inhibit both pathways and lead to a significant reduction in *I*_*SC*_ in human airway epithelia. Interestingly, in primary HBEs, Ca^2+^-dependent *I*_*SC*_ was completely abolished; thus, they may be particularly sensitive to e-vapour. Although 42 of 100 flavoured e-liquids were reported to increase cytosolic Ca^2+^ in Calu-3 cells [41], we did not observe an increase in Ca^2+^ for the three e-vapour brands we tested (data not shown). Therefore, we determined whether exposure to e-vapour affected ATP- or UTP-induced Ca^2+^ changes and ion transport changes in different cells.

The decrease in *I*_*SC*_ could be due to a decrease in proteins associated with Ca^2+^-dependent Cl^−^ secretion (TMEM16A, KCNN4), cAMP-dependent Cl^−^ secretion (CFTR, KCNQ1), and/or NKCC1, the Cl^−^ transporter. However, western blot analysis of these proteins did not support this hypothesis, suggesting that changes in ion channel or transporter protein expression do not explain the inhibitory effect of e-cigarettes. Because e-vapours decrease the important intracellular messengers, Ca^2+^ and cAMP, thereby reducing the transepithelial ion transport, further research is needed to elucidate the mechanism that underlies this reduction after e-vapour exposure. E-vapour exposure may cause ion channel dysfunction, resulting in reduced ion transport. In mice, CFTR and Ca^2+^-activated K^+^ (BK) channel activity is impaired by PG/VG aerosols containing Nic [42], and in primary HBEs, BK channel activity is inhibited by PG aerosols [43]. A reduction in membrane fluidity caused by VG aerosols preferentially affects CFTR rather than CaCC activity in primary HBEs [6]. E-vapour toxins, including reactive aldehydes such as acrolein, may inhibit CFTR function [44]. Although inhaling Nic in the form of aerosols reduces CFTR directly and undermines the mucociliary response to inhaled pathogens [7], we found a similar reduction in Ca^2+^, cAMP levels, and in the increases of *I*_*SC*_ for e-vapour with and without Nic. Notably, prior studies have not measured intracellular Ca^2+^ or cAMP. Thus, our data provide a possible mechanism for the reduction in channel activities. Although we saw no changes in the expression of various ion channels or transporters in primary HBEs or 16HBE14o- and Calu-3 cells (data not shown), a proteomic study of e-cigarette vapers with normal lung function showed that chronic e-cigarette use alters more than 200 proteins in lung epithelia. As we used PG/VG (the major solvent of various e-liquids) as a control, the detrimental effects of different e-vapours can be attributed to their flavours.

Our study has certain limitations. In our experiments, the epithelia were transferred from the incubation chamber to the Ussing chamber for calcium or cAMP and *I*_*SC*_ measurements, and 5–10 min passed between the end of the exposure and the beginning of the calcium or cAMP and *I*_*SC*_ measurement. Consequently, we were not able to expose the epithelial cells to CS or e-vapour at the time of intracellular calcium or cAMP measurement. Moreover, as e-liquids are autofluorescent, we were unable to detect instant real-time changes in intracellular calcium and cAMP in response to CS or e-vapour. Moreover, we have not addressed the question regarding the chemical composition of e-cigarette emissions, particularly in relation to power settings (30 W vs. 70 W), as quantifying emissions is essential for contextualising experimental outcomes. E-cigarettes have cumulative effects on the airway epithelium of real-world smokers. Thus, this study based on acute exposure to an in vitro ALI model is limited in its conclusions. In future studies, experimental methods of simulating chronic e-cigarette exposure should be developed, and real smokers’ airway epithelium samples should be used to study long-term effects. In addition, we were unable to detect changes in intracellular calcium or cAMP in real time during exposure to CS or e-vapour. To determine whether any agonist-induced changes in intracellular calcium or cAMP are due to e-vapour and not its major solvent (PG/VG), we employed PG/VG as a more appropriate e-vapour control. However, the flavouring tested herein was from three sources only, and the changes caused by flavouring exposure are therefore not applicable to all flavours currently on the market. Finally, the experimental data obtained from single donor–derived primary cells may not be representative and reproducible, and larger sample sizes should be used in the future. The ALI-HBE model used in this study mimicked the structure and physiological function of human bronchial epithelium to a certain extent; however, in vivo experiments are still required to confirm the experimental results and conclusions.

In summary, we determined the effects of e-cigarettes on ion transport activities in two commonly used human bronchial epithelial cell lines (16BHE14o- and Calu-3) and fully differentiated primary human airway epithelia. Exposure of human airway epithelia to e-vapour, with or without Nic, resulted in Ca^2+^- and cAMP-dependent ion transport dysfunction that may predispose the smoker to vaping-related lung injury. These results provide important new information because e-liquids are perceived to be safer alternatives to cigarettes. Further, we measured only the acute effects of e-vapour exposure on the airway epithelium, whereas the longer-term exposures of regular e-cigarette users may be even more detrimental to ion transport. Further research in animal models or human vapers is needed to determine the long-term effects of e-liquids on ion transport.

## Data Availability

No datasets were generated or analysed during the current study.

## Abbreviations

CaCC: Calcium-activated Cl^−^ channels
CFTR: Cystic fibrosis transmembrane conductance regulator
CS: Cigarette smoke
CFP: Cyan Fluorescent Protein
cAMP: Cyclic AMP
KCNQ1: cAMP-dependent K^+^ channel
e-cigarettes: Electronic cigarettes
e-vapour: E-cigarette vapour
Epac: Exchange protein directly activated by cAMP
FRET: Fluorescence resonance energy transfer
HBEs: Human bronchial epithelial cells
[Ca^2+^]_i_: Intracellular calcium concentration
NKCC1: Na^+^/K^+^/2Cl^−^ cotransporter
Nic: Nicotine
PG/VG: Propylene glycol/vegetable glycerine
PVDF: Polyvinylidene difluoride
*I*_*SC*_: Short-circuit current
TMEM16A: Transmembrane protein 16A
TPM: Total particulate matter
KCNN4: Ca^2+^-activated K^+^ channel
YFP: Yellow Fluorescent Protein

## References

[1] Cantin AM et al (2006) Cystic fibrosis transmembrane conductance regulator function is suppressed in cigarette smokers. Am J Respir Crit Care Med 173(10):1139–1144 16497995 10.1164/rccm.200508-1330OC

[2] Patel W et al (2018) Increases in cytosolic Ca^2+^ induce dynamin- and calcineurin-dependent internalisation of CFTR. Cell Mol Life Sci 76(5):977–994 30547226 10.1007/s00018-018-2989-3 PMC6394554

[3] Henke K et al (2023) 30-min exposure to tobacco smoke influences airway ion transport-an in vitro study. Curr Oncol 30(7):7007–7018 37504368 10.3390/curroncol30070508 PMC10378258

[4] Crotty Alexander LE et al (2018) Chronic inhalation of e-cigarette vapor containing nicotine disrupts airway barrier function and induces systemic inflammation and multiorgan fibrosis in mice. Am J Physiol Regul Integr Comp Physiol 314(6):R834–R847 29384700 10.1152/ajpregu.00270.2017 PMC6032308

[5] Raduka A et al (2023) Electronic cigarette exposure disrupts airway epithelial barrier function and exacerbates viral infection. Am J Physiol Lung Cell Mol Physiol 325(5):L580–L593 37698113 10.1152/ajplung.00135.2023 PMC11068398

[6] Kim MD et al (2022) Vegetable glycerin e-cigarette aerosols cause airway inflammation and ion channel dysfunction. Front Pharmacol 13:1012723 36225570 10.3389/fphar.2022.1012723 PMC9549247

[7] Rasmussen LW et al (2023) Nicotine aerosols diminish airway CFTR function and mucociliary clearance. Am J Physiol Lung Cell Mol Physiol 324(5):L557–L570 36852921 10.1152/ajplung.00453.2021 PMC10085557

[8] Cai MY et al (2022) Role of carbon monoxide in oxidative stress-induced senescence in human bronchial epithelium. Oxid Med Cell Longev 2022:5199572 36193088 10.1155/2022/5199572 PMC9526622

[9] Wong AM et al (2009) Apical versus basolateral P2Y_6_ receptor-mediated Cl^-^ secretion in immortalized bronchial epithelia. Am J Respir Cell Mol Biol 40(6):733–745 19011163 10.1165/rcmb.2008-0020OC

[10] Manevski M et al (2022) E-cigarette synthetic cooling agent WS-23 and nicotine aerosols differentially modulate airway epithelial cell responses. Toxicol Rep 9:1823–1830 36518432 10.1016/j.toxrep.2022.09.010 PMC9742947

[11] Antherieu S et al (2017) Comparison of cellular and transcriptomic effects between electronic cigarette vapor and cigarette smoke in human bronchial epithelial cells. Toxicol In Vitro 45(Pt 3):417–425 28065790 10.1016/j.tiv.2016.12.015

[12] Kesimer M (2019) Another warning sign: high nicotine content in electronic cigarettes disrupts mucociliary clearance, the essential defense mechanism of the lung. Am J Respir Crit Care Med 200(9):1082–1084 31199664 10.1164/rccm.201905-1080ED PMC6888647

[13] Hao Y et al (2014) P2Y_6_ receptor-mediated proinflammatory signaling in human bronchial epithelia. PLoS ONE 9(9):e106235 25243587 10.1371/journal.pone.0106235 PMC4171090

[14] van der Krogt GN et al (2008) A comparison of donor-acceptor pairs for genetically encoded FRET sensors: application to the Epac cAMP sensor as an example. PLoS ONE 3(4):e1916 18382687 10.1371/journal.pone.0001916 PMC2271053

[15] Zhang RG et al (2019) Anti-inflammatory action of HO-1/CO in human bronchial epithelium in response to cationic polypeptide challenge. Mol Immunol 105:205–212 30553057 10.1016/j.molimm.2018.12.002

[16] Zhang RG et al (2020) b_2_ adrenoceptor signaling regulates ion transport in 16HBE14o- human airway epithelial cells. J Cell Physiol 235(11):8387–8401 32239700 10.1002/jcp.29683

[17] Communi D et al (1999) Expression of P2Y receptors in cell lines derived from the human lung. Br J Pharmacol 127(2):562–568 10385259 10.1038/sj.bjp.0702560 PMC1566032

[18] Ito Y et al (2004) ATP release triggered by activation of the Ca^2+^-activated K^+^ channel in human airway Calu-3 cells. Am J Respir Cell Mol Biol 30(3):388–395 12947021 10.1165/rcmb.2003-0184OC

[19] Cozens AL et al (1994) CFTR expression and chloride secretion in polarized immortal human bronchial epithelial cells. Am J Respir Cell Mol Biol 10(1):38–47 7507342 10.1165/ajrcmb.10.1.7507342

[20] Forbes B (2000) Human airway epithelial cell lines for in vitro drug transport and metabolism studies. Pharm Sci Technolo Today 3(1):18–27 10.1016/s1461-5347(99)00231-x 10637597

[21] Forbes B et al (2003) The human bronchial epithelial cell line 16HBE14o- as a model system of the airways for studying drug transport. Int J Pharm 257(1–2):161–167 12711171 10.1016/s0378-5173(03)00129-7

[22] Jeulin C et al (2008) EGF mediates calcium-activated chloride channel activation in the human bronchial epithelial cell line 16HBE14o-: involvement of tyrosine kinase p60c-src. Am J Physiol Lung Cell Mol Physiol 295(3):L489–L496 18586953 10.1152/ajplung.90282.2008

[23] Wan H et al (2000) Tight junction properties of the immortalized human bronchial epithelial cell lines Calu-3 and 16HBE14o. Eur Respir J 15(6):1058–1068 10885425 10.1034/j.1399-3003.2000.01514.x

[24] Grumbach Y et al (2009) LXA4 stimulates ZO-1 expression and transepithelial electrical resistance in human airway epithelial (16HBE14o-) cells. Am J Physiol Lung Cell Mol Physiol 296(1):L101–L108 18849442 10.1152/ajplung.00018.2008

[25] Lipsa D et al (2016) Inflammatory effects induced by selected limonene oxidation products: 4-OPA, IPOH, 4-AMCH in human bronchial (16HBE14o-) and alveolar (A549) epithelial cell lines. Toxicol Lett 262:70–79 27575568 10.1016/j.toxlet.2016.08.023

[26] Chow AW et al (2010) Polarized secretion of interleukin (IL)-6 and IL-8 by human airway epithelia 16HBE14o- cells in response to cationic polypeptide challenge. PLoS ONE 5(8):e12091 20711426 10.1371/journal.pone.0012091 PMC2920803

[27] Parilla NW et al (2006) CpG DNA modulates interleukin 1beta-induced interleukin-8 expression in human bronchial epithelial (16HBE14o-) cells. Respir Res 7(1):84 16740161 10.1186/1465-9921-7-84 PMC1489942

[28] Below S et al (2009) Virulence factors of Staphylococcus aureus induce Erk-MAP kinase activation and c-Fos expression in S9 and 16HBE14o- human airway epithelial cells. Am J Physiol Lung Cell Mol Physiol 296(3):L470–L479 19098123 10.1152/ajplung.90498.2008

[29] Abraham G et al (2004) Expression of functional b_2_-adrenergic receptors in the lung epithelial cell lines 16HBE14o-, Calu-3 and A549. Biochim Biophys Acta 1691(2–3):169–179 15110997 10.1016/j.bbamcr.2004.02.002

[30] Missiaen L et al (2002) Ca^2+^ uptake and release properties of a thapsigargin-insensitive nonmitochondrial Ca^2+^ store in A7r5 and 16HBE14o- cells. J Biol Chem 277(9):6898–6902 11861657 10.1074/jbc.M110939200

[31] Effah F et al (2023) Toxicological assessment of E-cigarette flavored E-liquids aerosols using Calu-3 cells: A 3D lung model approach. Toxicology 500:153683 38013136 10.1016/j.tox.2023.153683 PMC10826471

[32] Ghosh A et al (2017) Little cigars are more toxic than cigarettes and uniquely change the airway gene and protein expression. Sci Rep 7:46239 28447619 10.1038/srep46239 PMC5406835

[33] Shan J et al (2011) Anion secretion by a model epithelium: more lessons from Calu-3. Acta Physiol (Oxf) 202(3):523–531 21251238 10.1111/j.1748-1716.2011.02253.x

[34] Bui CHT et al (2019) Tropism of influenza B viruses in human respiratory tract explants and airway organoids. Eur Respir J 54(2):1900008 31097520 10.1183/13993003.00008-2019

[35] Chambers LA et al (2007) Liquid movement across the surface epithelium of large airways. Respir Physiol Neurobiol 159(3):256–270 17692578 10.1016/j.resp.2007.06.005 PMC2696130

[36] Tarran R et al (2006) Regulation of normal and cystic fibrosis airway surface liquid volume by phasic shear stress. Annu Rev Physiol 68:543–561 16460283 10.1146/annurev.physiol.68.072304.112754

[37] Danahay H, Jackson AD (2005) Epithelial mucus-hypersecretion and respiratory disease. Curr Drug Targets Inflamm Allergy 4(6):651–664 17305521 10.2174/156801005774912851

[38] Boucher RC (2002) An overview of the pathogenesis of cystic fibrosis lung disease. Adv Drug Deliv Rev 54(11):1359–1371 12458149 10.1016/s0169-409x(02)00144-8

[39] Bardou O et al (2009) Molecular diversity and function of K^+^ channels in airway and alveolar epithelial cells. Am J Physiol Lung Cell Mol Physiol 296(2):L145–L155 19060226 10.1152/ajplung.90525.2008

[40] Caputo A et al (2008) TMEM16A, a membrane protein associated with calcium-dependent chloride channel activity. Science 322(5901):590–594 18772398 10.1126/science.1163518

[41] Rowell TR et al (2020) Flavored e-liquids increase cytoplasmic Ca^2+^ levels in airway epithelia. Am J Physiol Lung Cell Mol Physiol 318(2):L226–L241 31693394 10.1152/ajplung.00123.2019 PMC7052665

[42] Garcia-Arcos I et al (2016) Chronic electronic cigarette exposure in mice induces features of COPD in a nicotine-dependent manner. Thorax 71(12):1119–1129 27558745 10.1136/thoraxjnl-2015-208039 PMC5136722

[43] Kim MD et al (2023) E-cigarette aerosols of propylene glycol impair BK channel activity and parameters of mucociliary function. Am J Physiol Lung Cell Mol Physiol 324(4):L468–L479 36809074 10.1152/ajplung.00157.2022 PMC10042605

[44] Lin VY et al (2018) Vaporized E-cigarette liquids induce ion transport dysfunction in airway epithelia. Am J Respir Cell Mol Biol 61(2):162–173 10.1165/rcmb.2017-0432OC PMC6670031 30576219

